# Differential involvement of *RASSF2* hypermethylation in breast cancer subtypes and their prognosis

**DOI:** 10.18632/oncotarget.4062

**Published:** 2015-06-04

**Authors:** Noemi Perez-Janices, Idoia Blanco-Luquin, Natalia Torrea, Therese Liechtenstein, David Escors, Alicia Cordoba, Francisco Vicente-Garcia, Isabel Jauregui, Susana De La Cruz, José Juan Illarramendi, Valle Coca, Maria Berdasco, Grazyna Kochan, Berta Ibañez, José Miguel Lera, David Guerrero-Setas

**Affiliations:** ^1^ Cancer Epigenetics Group, Navarrabiomed-Fundación Miguel Servet (FMS), Instituto de Investigaciones Sanitarias de Navarra-IdiSNA, Navarra, Spain; ^2^ Division of Infection and Immunity, Rayne Institute, University College London (UCL), London, United Kingdom; ^3^ Cancer Immunomodulation Group, Navarrabiomed-Fundacion Miguel Servet, IdiSNA, Navarra, Spain; ^4^ Wellcome Trust Centre for Cell Biology, University of Edinburgh, Edinburgh, United Kingdom; ^5^ Department of Pathology, Complejo Hospitalario de Navarra, Navarra Health Service, Pamplona, Navarra, Spain; ^6^ Department of Surgery, Complejo Hospitalario de Navarra, Navarra Health Service, Navarra, Spain; ^7^ Department of Medical Oncology, Complejo Hospitalario de Navarra, Navarra Health Service, Navarra, Spain; ^8^ Biobank Unit, Navarrabiomed-Fundacion Miguel Servet, IdiSNA, Navarra, Spain; ^9^ Cancer Epigenetics Group, Cancer Epigenetics and Biology Programme (PEBC), Bellvitge Biomedical Research Institute (IDIBELL), Barcelona, Spain; ^10^ Red de Evaluación en Servicios Sanitarios y Enfermedades Cronicas (REDISSEC), Navarrabiomed-Fundación Miguel Servet, IdiSNA, Navarra, Spain

**Keywords:** breast cancer, DNA methylation, prognosis, RASSF1, RASSF2

## Abstract

Breast cancer is a heterogeneous disease that can be subdivided into clinical, histopathological and molecular subtypes (luminal A-like, luminal B-like/HER2-negative, luminal B-like/HER2-positive, HER2-positive, and triple-negative). The study of new molecular factors is essential to obtain further insights into the mechanisms involved in the tumorigenesis of each tumor subtype. *RASSF2* is a gene that is hypermethylated in breast cancer and whose clinical value has not been previously studied. The hypermethylation of *RASSF1* and *RASSF2* genes was analyzed in 198 breast tumors of different subtypes. The effect of the demethylating agent 5-aza-2′-deoxycytidine in the re-expression of these genes was examined in triple-negative (BT-549), HER2 (SK-BR-3), and luminal cells (T-47D). Different patterns of RASSF2 expression for distinct tumor subtypes were detected by immunohistochemistry. *RASSF2* hypermethylation was much more frequent in luminal subtypes than in non-luminal tumors (*p* = 0.001). The re-expression of this gene by lentiviral transduction contributed to the differential cell proliferation and response to antineoplastic drugs observed in luminal compared with triple-negative cell lines. *RASSF2* hypermethylation is associated with better prognosis in multivariate statistical analysis (*P* = 0.039). In conclusion, *RASSF2* gene is differently methylated in luminal and non-luminal tumors and is a promising suppressor gene with clinical involvement in breast cancer.

## INTRODUCTION

Breast cancer is a multifactorial disease arising from the combined effects of genetic and environmental factors, and may be hereditary or sporadic depending on the predominant factors [1]. Its treatment and prognosis differ according to the specific ontogeny of the cancer cells. Thus, breast tumors have been classified into four categories (luminal A, luminal B, Her2 and triple-negative) on the basis of the immunohistochemical detection of estrogen receptor (ER), progesterone receptor (PR) and epidermal growth factor type II receptor (HER2). Ki-67 was subsequently included as a new marker to broaden this classification to five subgroups: luminal A (ER- and/or PR-positive, HER2-negative and Ki-67 < 14%), luminal B (ER- and/or PR-positive, HER2-negative and Ki-67 ≥ 14%), luminal-HER2 (ER- and/or PR-positive, HER2-positive), HER2 (ER- and/or PR-negative; HER2-positive) and triple-negative patients (ER- and PR-negative, HER2-negative) [2]. These subtypes differ pathologically and clinically. For example, luminal A and triple-negative patients are characterized by better and worse prognosis, respectively. A new classification of breast tumors into luminal A-like, luminal B-like/HER2-negative, luminal B-like/HER2-positive, HER2-positive and triple-negative tumors [3] has recently been proposed that includes the percentage of PR cells as a classification marker; some reports have been published that have adopted this new classification [4]. Molecular alterations help to characterize more aggressive tumors belonging to the same and different subtypes, being the triple-negative subtype the most aggressive one as it was confirmed in a multi-centric study [5].

The mitogen-activated extracellular signal-regulated kinase (MEK) pathway is one of the best-characterized kinase cascades triggered in cancer by growth factors or activating mutations of major oncogenic proteins in this pathway, e.g., Ras and Raf [6]. In breast cancer, Ras signaling is also altered but mutations of the Ras gene are uncommon [6]. Epigenetic alterations of effectors of this gene, such as RASSF1 Ras association (RalGDS/AF-6) domain family member 1 (*RASSF1*) [7], are very frequent in this type of cancer [8, 9], although their role in tumorigenesis remains to be elucidated.

*RASSF2* is another member of the RASSF family that has been little studied in breast cancer [10]. The encoding gene is located at the *20p13* chromosomal position and contains 12 exons that give rise to a 326-aminoacid protein with important functions as an apoptosis inductor and cell growth inhibitor [11, 12]. RASSF2 is expressed in many tissues, and the hypermethylation of its promoter has been described in colorectal, gastric, Ewing sarcoma and nasopharyngeal cancers, among others [11–19]. This gene is also hypermethylated in breast cancer [20], but no additional studies of its clinical involvement in breast cancer subtypes have been performed in large groups until now. Conversely, there are many reports about the possible role of *RASSF1* hypermethylation as a biomarker in breast cancer related to pathological characteristics of worse prognosis [9], but almost nothing is known about its involvement in breast cancer subtypes.

Our objectives were to analyze the presence of methylation of the less-studied *RASSF2* gene in breast cancer subtypes, along with the well-known gene *RASSF1*, and to evaluate the prognostic role of these alterations in patients. Breast cancer cell lines were treated with the demethylating agent 5-aza-2′-deoxycytidine (5-azadC) to study the effect of restoring RASSF2 expression by lentivector technology on cell proliferation and the response to treatment in luminal and triple-negative breast cancer cell lines.

## RESULTS

### *RASSF1* and *RASSF2* genes are hypermethylated in breast cancer subtypes

**Figure 1:**
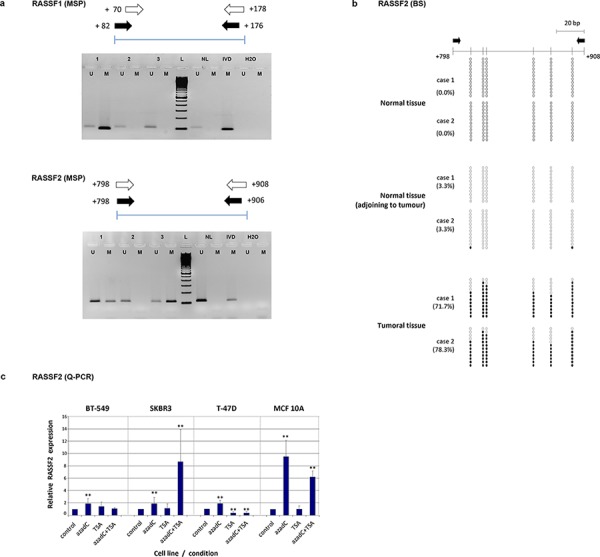
**a.** Methylation-specific PCR (MSP) for methylation analysis of *RASSF1* and *RASSF2* genes in tumor samples. Schemes of the region analyzed by this technique and images from different tumours including unmethylated (U) and methylated (M) cases are shown for each gene(NL: DNA from normal lymphocytes; IVD: *in vitro*-methylated DNA). **b.** Bisulfite-sequencing analysis methylation status in 12 clones containing the *RASSF2* sequence from two example cases of normal breast tissue from reduction mammoplasties, normal tissue adjacent to a tumor, and tumoral tissue. The methylation status of analyzed CpG sites is shown for each clone (open and filled circles represent unmethylated and methylated clones, respectively). Black arrows indicate the location of the MSP primers. The location of each CpG site relative to the transcription initiation site is shown by a vertical bar. **c.** Expression level of RASSF2 by quantitative PCR (Q-PCR) in breast cancer cell lines treated with 5-azadC and/or trichostatin. The relative level of expression was compared with the untreated cells (control), whose value was taken as 100%. Bars represent mean ± standard deviation of the relative expression level of three experiments. ** indicates very statistically significant (*p* < 0.01) differences in gene expression between treated and untreated cells.

*RASSF1* and *RASSF2* were almost 100% hypermethylated in all cell lines. In the tumors, the *RASSF1* and *RASSF2* genes could be analyzed by methylation-specific PCR (MSP) in 143 (72.2%) and 168 (84.8%) cases, respectively (Table 1), yielding percentages of hypermethylation of 74.1% and 66.7%, respectively (Figure 1a). The percentage DNA methylation of these genes was not associated with the age of the patients (*p* = 0.283 for *RASSF1*; *p* = 0.721 for *RASSF2*) or with lymph node involvement at the time of diagnosis (*p* = 0.811 for *RASSF1*; *p* = 0.540 for *RASSF2*).

**Table 1:** Demographic, pathological and molecular data of patients

		*n* (%)
***Demographic data***		
**Median age (range)**		56 (25–94)
***Pathological characteristics***		
**Tumor size**	Median (range)	1.6 (0.3, 15.0)
**Tumor location**	Right	95 (48%)
	Left	103 (52%)
**Histological grade**^1^	I	33 (17.2%)
	II	71 (37%)
	III	88 (45.4%)
**Histological type**	Ductal	144 (72.7%)
	Ductal (Tubular)	16 (8.1%)
	Ductal (Apocrine)	16 (8.1%)
	Ductal (Cribiform) Lobular	8 (4.0%) 14 (7.1%)
**LNI**^2^	Yes	79 (41.4%)
	No	112 (58.6%)
**Tumor subtype**^3^	Luminal A-like	32 (16.4%)
	Luminal B-like (HER2-negative)	58 (29.7%)
	Luminal B-like (HER2-positive)	47 (24.1%)
	HER2-positive	19 (9.7%)
	Triple-negative	39 (20.0%)
**Luminal subtype**	Yes	137 (70.3%)
	No	58 (29.7%)
***Gene methylation***		
***RASSF1* methylation**	Yes	106 (74.1%)
	No	37 (25.9%)
***RASSF2* methylation**	Yes	112 (66.7%)
	No	56 (33.3%)

1 Grade according to the World Health Organization (WHO) grading system;

2 lymph node involvement;

3 Classification following St Gallen International Expert Consensus on the Primary Therapy of Early Breast Cancer 2013, published in Ann Oncol 2013; 24: 2206–2223

*RASSF2* hypermethylation differed between tumor subtypes as defined by the St Gallen 2013 criteria (i.e., luminal A-like, luminal B-like/HER2-negative, luminal B-like/HER2-positive, HER2-positive, and triple-negative) (*p* = 0.005) (Table 2). It is notable that the highest percentage methylation was present in luminal subtypes (24 methylated cases out of 50 luminal tumors; 48.0%) compared with non-luminal tumors (30 methylated cases out of 118 tumors; 25.4%) (*p* = 0.001). These results were also found in the subtypes defined by the 2011 St Gallen criteria (i.e., luminal A, luminal B, luminal-HER2, HER2 and triple-negative) (*p* < 0.001; data not shown). *RASSF1* gene hypermethylation was also differently distributed with respect to the luminal tumor subtypes, although the pattern was less significant (*p* = 0.046). The clear association of this alteration with luminal and non-luminal subtypes found for *RASSF2* was not found for *RASSF1* (*p* = 0.083). Therefore, we considered it more interesting to analyze in greater depth the less well studied hypermethylation of *RASSF2* rather than that of *RASSF1*.

**Table 2:** Association of gene hypermethylation with pathological diagnosis, vascular invasion and histological type

	*RASSF1 hypermethylation*			*RASSF2 hypermethylation*		
	Yes *n* (%)	No *n* (%)	*p*^1^	Yes *n* (%)	No *n* (%)	*p*^1^
**Histological grade** **I** **II** **III**	19 (73.1) 38 (79.2) 46 (71.9)	7 (26.9) 10 (20.8) 18 (28.1)	0.726	23 (79.3) 40 (67.8) 45 (60.8)	6 (20.7) 19 (32.2) 29 (39.2)	0.196
**LNI^2^** **Yes** **No**	43 (72.9) 59 (74.7)	16 (27.1) 20 (25.3)	0.811	45 (69.2) 62 (64.6)	20 (30.8) 34 (35.4)	0.540
**Tumor subtype^3^** **Luminal A-like** **Luminal B-like (HER2-negative)** **Luminal B-like (HER2-positive)** **HER2-positive** **Triple-negative**	15 (65.2) 31 (81.6) 33 (82.5) 11 (84.6) 16 (55.2)	8 (34.5) 7 (18.4) 7 (17.5) 2 (15.4) 13 (44.8)	0.046	22 (81.5) 39 (79.6) 27 (64.3) 9 (56.2) 15 (44.1)	5 (18.5) 10 (20.4) 15 (35.7) 7 (43.8) 19 (55.9)	0.005
**Luminal subtype** **Yes** **No**	27 (64.3) 79 (78.2)	15 (35.7) 22 (21.8)	0.083	24 (48.0) 30 (25.4)	26 (52.0) 88 (74.6)	0.001

1 Probabilities associated with chi-square test (statistically significant values in bold);

2 LNI: lymph node involvement;

3 Classification following St Gallen International Expert Consensus on the Primary Therapy of Early Breast Cancer 2013, published in Ann Oncol 2013; 24: 2206–2223

*RASSF2* hypermethylation was confirmed by bisulfite sequencing (BS) in clones derived from normal tissue adjacent to the tumor, completely normal tissue and from tumor samples (Figure 1b). All breast cancer samples analyzed for BS were highly hypermethylated (from 60% to 78%) in all CpG islands. Normal breast tissue from mammoplasties showed no hypermethylation (0.0%) at any of the positions analyzed, and negligible levels (3.3%) in normal tissue adjacent to the tumor (Figure 1b). BS of cell lines confirmed the high percentage of hypermethylation of this gene (>90% in all the cell lines).

### Demethylating agents induce the re-expression of RASSF2

RT-PCR was performed to analyze RASSF2 expression in control and treated cell lines and in breast tumors. Relative expression per control and treated cell lines are shown in Table 3. The treatment with 5-azadC increased RASSF2 expression in all cell lines (*P* < 0.01) (Figure 1c), except for 5-azadC and TSA treatment of T-47D cells. The most remarkable increases were found for the treatment of 5-azadC and TSA treatments in SK-BR-3 cells, and for 5-azadC in MCF 10A cells, with respective 8-fold and 9-fold greater RASSF2 expression in treated versus control cells.

**Table 3:** Quantitative reverse-transcription PCR results in treated compared with control cell lines

	Cell line							
	BT-549		SK-BR-3		T-47D		MCF 10A	
**Treatment**	**2^−ΔΔCt^**^1^	***p***	**2^−ΔΔCt^**	***p***	**2^−ΔΔCt^**	***p***	**2^−ΔΔCt^**	***p***
*5-azadC*	1.88	< 0.001	1.87	0.004	1.88	< 0.001	9.53	< 0.001
*TSA*	1.46	0.051	1.11	0.680	0.39	< 0.001	1.02	0.896
*5-azadC + TSA*	1.03	0.867	8.63	<0.001	0.37	< 0.001	6.22	< 0.001

1 2^−ΔΔCt^ : Relative change in expression of genes of the treated cells compared with the control group

In pairwise comparisons of normal tumors from luminal (methylated) and triple-negative tumors (unmethylated) the expression of RASSF2 in the triple-negative subgroup was 1.87 times that in the luminal subgroup of tumors (data not shown).

**Figure 2:**
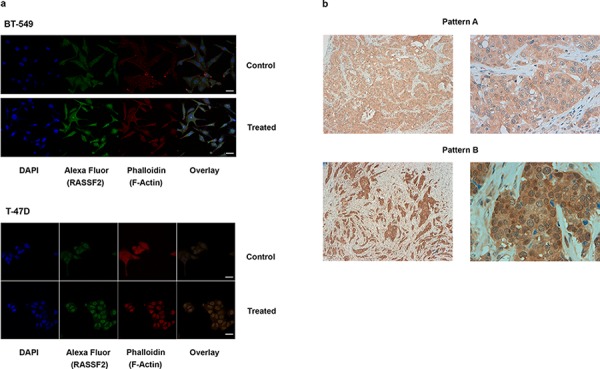
**a.** Immunofluorescence images of RASSF2 re-expression in BT-549 and T-47D cells (× 630), (bar scale = 25 μm)The target protein is indicated with Alexa Fluor 488 (green staining), and the cytoskeleton is marked with phalloidin. An increase in the expression of RASSF2 protein in the cytoplasm and nucleus is shown. **b.** IHC staining for a tumor positive for RASSF2 expression pattern A (up, right and left) and pattern B (down, right and left), unmethylated and methylated for this gene, respectively.

For IF analyses, cells exposed to the combination of 5-azadC and TSA treatment clearly showed RASSF2 re-expression compared with the control cells in all the cell lines tested (Figure 2a, Supplementary Table 1). This increase in expression was reflected in terms of total protein content, and also at cytoplasmic and nuclear locations (*p* < 0.001, in all cell lines).

For IHC, two predominant staining patterns for RASSF2 expression were found: cytoplasmic and nuclear. The cytoplasmic pattern consisted of a predominant expression within the cytoplasm, with low or zero intensity expression within the nucleus (Figure 2b, up). The nuclear pattern consisted of predominant RASSF2 expression in the nucleus with strong homogeneously intense expression in the cytoplasm (Figure 2b, down). Normal mammary ducts featured intense expression in the cytoplasm, with weaker expression in the nucleus (Supplementary Figure 1). It is notable that all the luminal tumors exhibited the cytoplasmic pattern whereas non-luminal tumors (HER2-positive, triple-negative tumors) showed cytoplasmic and nuclear patterns (*p* = 0.046). *RASSF2* hypermethylation was independent of these patterns (*p* = 0.550) and was more frequent in cases with low or absent RASSF2 expression (*P* = 0.061), with the majority of unmethylated cases strongly expressing RASSF2 protein (Supplementary Table 2).

### RASSF2 expression influences cell proliferation

**Figure 3:**
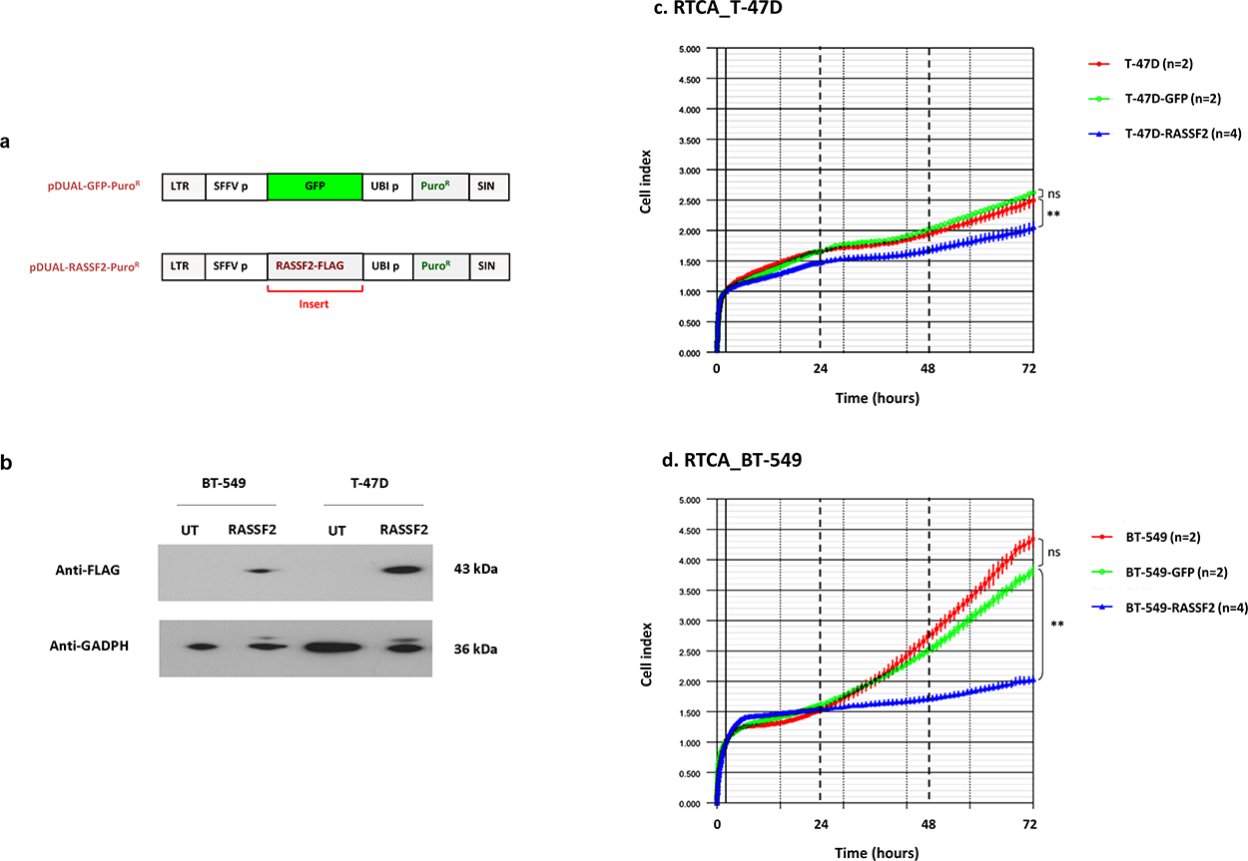
**a.** Schematic representation of pcDNA3.1/pDUAL encoding GFP (control) or RASSF2 and puromycin resistance. LTR: long tandem repeat; SFFV p: spleen focus-forming virus promoter; UBI p: ubiquitin promoter; Puro R: puromycin resistance; SIN: self-inactivating region. **b.** Western blot images using RASSF2-Flag-tag to detect RASSF2 in untransduced (UT) and transduced (RASSF2) BT-549 and T-47D cells. **c.** and **d.** Representative real-time cell monitoring (RTCA) results for control and transduced T-47D (c) and BT-549 cells (d), as indicated. Data is plotted as means of cell index with error bars (standard deviations) from duplicate cultures during the time of analysis. Relevant statistical comparisons are indicated with ** for very significant (*P* < 0.01) differences.

To gain insight into the role of RASSF2 in cancer cells of different subtypes, T-47D (luminal cells) and BT-549 (triple-negative cells) were transduced with GFP lentiviral vector (control cell lines) and with GFP lentiviral vector expressing a FLAG-tagged RASSF gene (Figure 3a). The restoration of RASSF2 expression was demonstrated in both cell lines by western blot with anti-FLAG antibody (Figure 3b).

Cell proliferation was monitored in control cells (not transduced and transduced just with GFP lentivector) and RASSF2-transduced cells by Real Time Cell Analysis (RTCA). Cell growth differed between T47 D RASSF2-GFP transduced and control cell lines (GFP-cells) (Figure 3c) (*p* < 0.010), being this change much more prominent in the case of BT-549 cells (Figure 3d) (*p* < 0.010).

### RASSF2 expression influences chemotherapy sensitivity

With regard to the effects of chemotherapy, T-47D and T-47D-RASSF2 cell lines were similarly sensitive to chemotherapy for all treatments and concentrations (Supplementary Table 3). These results suggest that RASSF2 may alter cell growth rather than the sensitivity to chemotherapy depending on the cell type. Interestingly, BT-549 and BT-549-RASSF2 presented an 11.1% difference in survival for treatment with low-dose cisplatin and 7.5% for low-dose cisplatin combined with a low dose of paclitaxel, but high doses of cisplatin were equally toxic for normal and RASSF2 cells. The results concerning cell response to low doses of cisplatin make this approach to be an interesting candidate for further studies.

### RASSF2 hypermethylation is associated with longer OS of breast cancer patients

**Figure 4:**
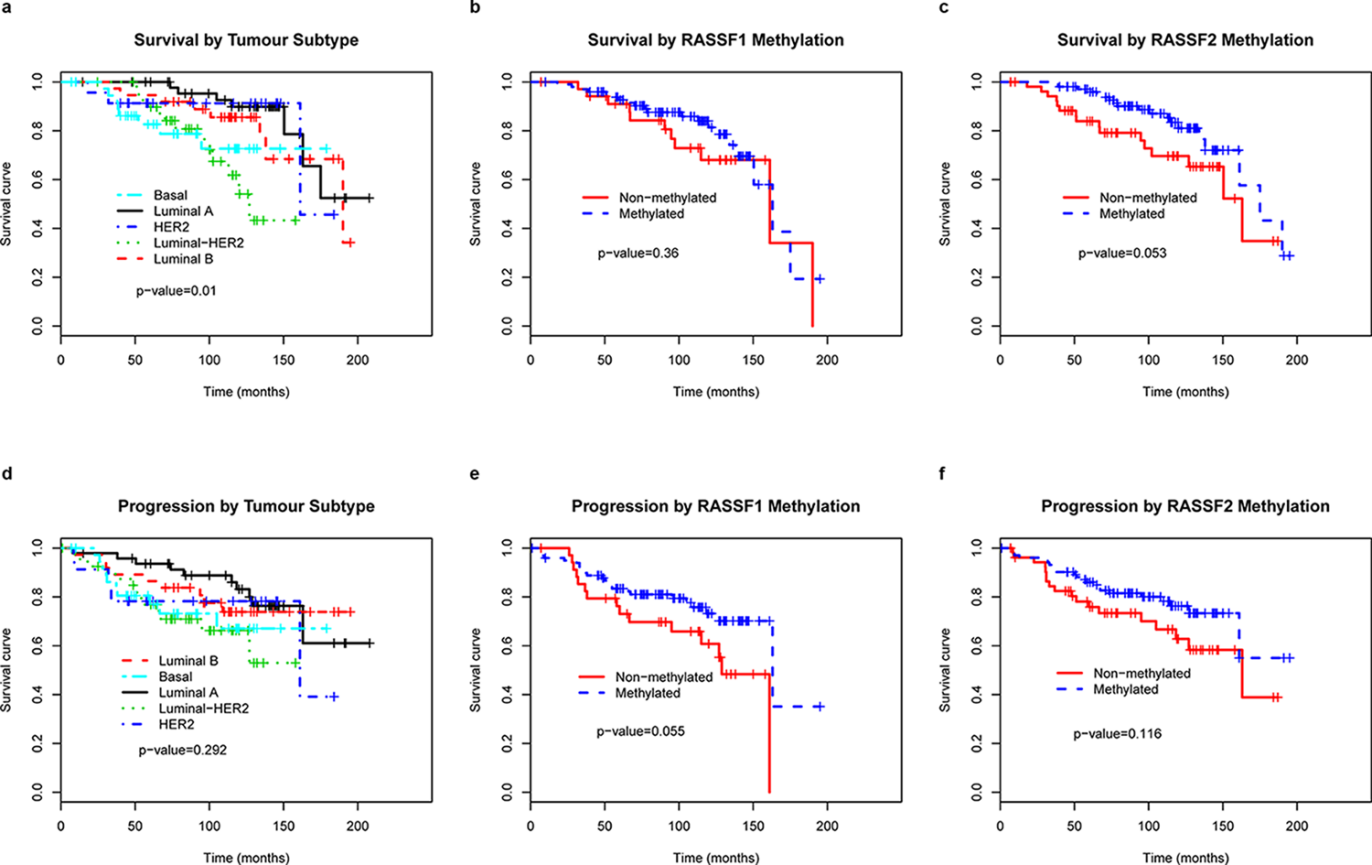
Kaplan–Meier curves predicting the probability of death a, b, c and progression d, e, f  in patients with breast cancer, by tumor subtype and *RASSF1* and *RASSF2* methylation status

To determine whether any of the variables were of prognostic significance, the survival of all the patients was analyzed. The univariate analysis confirmed that known factors such as tumor size, lymph node involvement and unfavorable histological grade were associated with worse prognosis, as previously demonstrated [1] (Table 4). Luminal-A like subtype and *RASSF2* hypermethylation were of better prognostic significance with regards to OS (*p* = 0.015, *p* = 0.053, respectively) (Figure 4). The patients with *RASSF2* hypermethylation showed longer PFS periods but without signification (*P* = 0.073). The multivariate analysis showed lymph node involvement and *RASSF2* hypermethylation to be associated with shorter and longer OS, respectively, independent of other factors (*p* = 0.021, *p* = 0.039, respectively) (Table 5). The statistical significance of the PFS and OS was greater in the multivariate analysis (*p* = 0.059 for DFS; *p* = 0.039 for OS) than in univariate tests (*p* = 0.073 and *p* = 0.053, respectively).

**Table 4:** Univariate Kaplan–Meier analysis of the risk of recurrence or death related to pathological and molecular variables in patients with breast cancer

		Progression		Overall survival	
Variable		HR (95% CI)^1^	*p*	HR (95% CI)	*p*
**Age**		1.016 (0.99–1.04)	0.210	1.01 (0.98–1.03)	0.580
**Tumor size**		1.28 (1.1–1.49)	0.004	1.19 (1.04–1.36)	0.010
**LNI**^2^	No	Ref.	<0.001	Ref.	<0.001
	Yes	3.2 (1.76–5.82)		3.32 (1.69–6.51)	
**Histological grade**	I	Ref.	0.047	Ref.	0.036
	II	1.27 (0.46–3.54)		5.65 (0.74–43.04)	
	III	2.45 (0.94–6.36)		9.74 (1.31–72.36)	
**Tumor subtype**^3^	Luminal A-like	Ref.	0.170	Ref.	0.015
	Luminal B-like (HER2-negative)	1.1 (0.43–2.82)		1.56 (0.49–4.95)	
	Luminal B-like (HER2-positive)	2.49 (0.99–6.23)		4.89 (1.55–15.37)	
	HER2-positive	1.26 (0.36–4.29)		1.29 (0.23–7.14)	
	Triple-negative	2.06 (0.78–5.46)		3.93 (1.16–13.34)	
**Luminal subtype**	No	Ref.	0.520	Ref.	0.470
	Yes	0.81 (0.43–1.52)		0.77 (0.37–1.59)	
***RASSF1* hypermethylation**	No	Ref.	0.063	Ref.	0.360
	Yes	0.53 (0.27–1.01)		0.71 (0.34–1.49)	
***RASSF2* hypermethylation**	No	Ref.	0.073	Ref.	0.053
	Yes	0.57 (0.31–1.05)		0.49 (0.25–0.95)	

1 CI, confidence interval; HR: hazard ratio;

2 LNI: lymph node involvement;

3 Classification following St Gallen International Expert Consensus on the Primary Therapy of Early Breast Cancer 2013, published in Ann Oncol 2013; 24: 2206–2223 (Ref., reference value)

**Table 5:** Multivariate Cox proportional analysis for determining disease outcome based on the risk of progression or death related to pathological and molecular variables, adjusted by age and tumor grade

		Progression		Overall survival	
Variable		HR (95% CI)	*p*	HR (95% CI)	*p*
**Tumor size**		1.24 (1.00–1.52)	0.045	1.07 (0.89–1.29)	0.046
**LNI**	No	Ref.	0.009	Ref.	0.021
	Yes	2.60 (1.27–5.32)		2.69 (1.16–6.24)	
**Histological grade**	I	Ref.	0.540	Ref.	0.370
	II	0.88 (0.26–3.00)		2.15 (0.26–17.94)	
	III	1.34 (0.40–4.53)		3.35 (0.39–28.49)	
**Tumor subtype**	Luminal A-like	Ref.	0.580	Ref.	0.680
	Luminal B-like (HER2-negative)	1.31 (0.42–4.04)		1.11 (0.28–4.37)	
	Luminal B-like (HER2-positive)	1.55 (0.49–4.94)		1.72 (0.43–6.86)	
	HER2-positive	0.53 (0.09–3.1)		0.41 (0.038–4.45)	
	Triple-negative	0.86 (0.22–3.40)		1.24 (0.25–6.13)	
***RASSF2* hypermethylation**	No	Ref.	0.059	Ref.	0.039
	Yes	0.49 (0.23–1.02)		0.41 (0.18–0.96)	

^1^CI, confidence interval; HR: hazard ratio; ^2^LNI: lymph node involvement; ^3^Classification following St Gallen International Expert Consensus on the Primary Therapy of Early Breast Cancer 2013, published in Ann Oncol 2013; 24: 2206–2223 (Ref., reference value)

## DISCUSSION

Breast cancers were classified on the basis of their histological appearances, which did not explain the heterogeneity observed in this type of cancer [21]. There are currently immunohistochemical markers that allow breast cancer to be stratified into clinically meaningful subgroups on the basis of pathological factors, including tumor size, histological grade, lymph node involvement, and clinically relevant predictive biomarkers, such as ER, PR, HER2 and Ki-67 expression [2]. Nevertheless, these immunohistochemical markers do not fully explain the great heterogeneity of breast cancer, making it necessary to analyze the prognostic role of new molecular candidates.

In our present study, *RASSF1* and *RASSF2* hypermethylation was very frequent in cell lines and tumors, but these alterations were not associated with each other. Hypermethylation of both genes was found to be independent of age, although this is known to contribute decisively to the methylation of some other genes [22]. In this study, *RASSF1* and *RASSF2* were hypermethylated in cell lines representative of tumor subtypes with best and worst prognosis, respectively [23]. MCF 10A cells also displayed methylation and although this immortalized mammary epithelial cell line is considered a normal cell line, several reports describe the presence of genetic alterations (copy number gain and *c-myc* amplification) [24] and epigenetic changes [25, 26] that suggest otherwise. The *RASSF2* gene is clearly regulated as this is derived from the fact that its re-expression is detected in all the methylated cell lines, including MCF 10A once it has been treated with a demethylating agent.

*RASSF1* was methylated in 74.1% of the patients, which indicates its value as a overall tumor marker for this disease, as described in breast and other types of cancer [9, 27]. In our study *RASSF1* hypermethylation varied between tumor subtypes, as observed in another study [35]. *RASSF2* was described as being a new putative tumor suppressor gene, given its role in growth inhibition, inactivated by hypermethylation in breast cancer [15]. Our group was the first to report this alteration in cervical and vulvar cancers [28, 29], and more recently in brain tumors, albeit in a small percentage of cases [30]. No further studies of the relevance of this alteration in different tumor subtypes and of the prognosis of patients with breast cancer have been performed until now.

In this study we found *RASSF2* hypermethylation to be frequent in breast cancer, and completely absent in breast normal tissue, corroborating its previously described cancer-specific role [20]. This alteration was clearly associated with luminal breast cancer subtypes (luminal A-like, luminal B-like/HER2-negative, luminal B-like/HER2-positive).

We have also confirmed that the pattern of expression of RASSF2 protein detected by IHC is different in luminal (predominantly cytoplasmic) and non-luminal (predominantly nuclear) tumors. These results suggest that RASSF2 translocates to the nucleus after reaching a certain level of cytoplasmic accumulation. This is of crucial importance given that the RASSF2 protein exerts its proapoptotic and cell cycle arrest functions in the nucleus, after interacting with protein kinase B (AKT) [20].

The cause of the clear association between *RASSF2* hypermethylation and luminal subtype remains to be determined, since the role of RAS signaling in the various subtypes of breast cancer has been little studied to date [31]. RASSF2 protein exerts its functions through K-RAS and phosphatidylinositol 3-kinase (PIK3CA). It would be interesting to investigate whether *RASSF2* hypermethylation is related to alterations in the PIK3CA pathway, since PIK3CA and RAS are pathways with apparently mutually exclusive alterations in breast cancer [32]. In colorectal cancer, *RASSF2* hypermethylation is associated with K-RAS mutations, which are essential in the progression of microsatellite-stable tumors. The inactivation of RASSF2 enhances K-RAS-mediated transformation, and overexpression of RASSF2 suppresses tumor cell growth, as described in another study [33]. This was also described in thyroid cancer cells, in which overexpression of RASSF2 reduced colony formation, because RASSF2 interacts with the proapoptotic kinases MST1 and MST2 and induces apoptosis in these cells [34]. In this report, the latest technology for monitoring cell growth has been used to highlight the different effect of RASSF2 expression in luminal and triple-negative cells, which has not been previously reported. In T-47D cells, RASSF2 re-expression slows down cell proliferation, whereas this effect is much more evident in transduced triple-negative BT-549 cells. This different effect possibly arises from the specific characteristics of cell growth and suppression of apoptosis in triple-negative tumors compared with luminal tumors [35–37]. It is important to note that the aberrant expression of certain proteins involved in Ras signaling is clearly present in the triple-negative subtype, as has just been demonstrated by proteomic analysis of cell lines [38]. Recent data also puts in evidence the different glycoproteomic profile of luminal and triple-negative tumors [39]. More studies of the role of RAS signaling in breast cancer progression are needed to address this issue [40].

In this study, frontline chemotherapy for breast cancer based on docetaxel and doxorubicin have demonstrated the same benefits for patients with a luminal subtype [41] as for triple-negative patients treated with paclitaxel preoperatively [42]. In this study, these treatments did not significantly influence *in vitro* cell survival with respect to the presence or absence of RASSF2 expression, in contrast to the positive effect of RASSF2 expression on the response to the regimen based on taxol and cisplatin in lung cancer [33]. Platinum-based chemotherapy has been considered as a candidate for the treatment of triple-negative breast cancer related to their BRCA1 phenotype [43], and our results suggest that cisplatin used alone or combined with other drugs could be used to treat triple-negative cells. As high doses of cisplatin are toxic independently of other factors, future experiments should focus on the response to low concentrations combined, or not, with other agents. Further *in vitro* studies in additional luminal and non-luminal cell lines are needed to determine the functional role of this protein in cell proliferation and response to treatment.

*RASSF2* hypermethylation was associated with a worse prognosis of gynecological cancer, as we had demonstrated before [44]. The worse prognosis associated with this alteration has also been described in nasopharyngeal carcinoma, gastric cancer, and Ewing sarcoma [19, 45, 46]. Conversely, in breast cancer, this alteration is a notable indicator of better prognosis that is independent of the tumor subtype and other prognostic factors.

In conclusion, to the best of our knowledge, this is the first report of *RASSF2* hypermethylation occurring preferentially in luminal tumors and having a good prognostic role for the patients. Further studies will help us determine how useful these alterations are for detecting and managing patients with poor prognosis.

## MATERIALS AND METHODS

### Cell lines

*In vitro* studies used luminal (T-47D, ATCC HTB-133), triple-negative (BT-549 ATCC HTB-122), and HER2 (SK-BR-3, ATCC CRL-10317) cells, and an immortalized mammary epithelial cell line (MCF 10A, ATCC HTB-30). Human embryonic kidney 293T cells were used for the lentivector experiments. BT-549 and T-47D were purchased from ATCC, whereas MCF 10A and 293T were kindly donated by Dr. Cos (University of Cantabria) and Dr. Escors (Navarrabiomed, FMS), respectively. T-47D, BT-549 and SK-BR-3 were cultured in RPMI-1640, supplemented with 1% penicillin/streptomycin and 10% fetal bovine serum (FBS) (Invitrogen, Life Technologies, Carlsbad, CA, USA). MCF-10A was cultured in DMEM/F-12 (Invitrogen, Life Technologies) supplemented with 1% penicillin/streptomycin, 10% FBS, 20 ng/mL of epidermal growth factor (EGF), 500 ng/mL of hydrocortisone and 10 μg/mL of insulin (Sigma-Aldrich, St Louis, MO, USA). 293T cells were cultured in DMEM supplemented with 1% penicillin/streptomycin and 10% FBS (Invitrogen, Life Technologies). Cells were grown in a humidified atmosphere (5% CO_2_, 37°C). Cells were always passaged before they reached confluence.

### Patients

The study group consisted of 198 patients diagnosed with primary infiltrating breast cancer between 2001 and 2007 in the Pathology Department of the Hospital de Navarra-Complejo Hospitalario de Navarra (Navarra Public Health System). All patients were operated upon and tumors were staged according to their size, histological grade, and lymph node involvement. None of the patients had received radiation or chemotherapy before surgery. The study was approved by the Regional Clinical Research Ethics Committee. The study also included 10 cases of normal tissue adjoining a breast tumor, and 5 cases of completely normal tissue obtained from reduction mammoplasties.

The diagnosis of these tumors was confirmed following microscopic inspection by a certified pathologist with expertise and dedication to breast pathology (A.C.). Tumors were classified into five subtypes (luminal A-like, luminal B-like/HER2-negative, luminal B-like/HER2-positive, HER2-positive and triple-negative) on the basis of previously established criteria [3]. Pathological and clinical characteristics are summarized in Table 1. The luminal (luminal A-like, luminal B-like/HER2-negative, luminal B-like/HER2-positive) and non-luminal (HER2-positive and triple-negative) groups comprised 135 (68.2%) and 63 (31.8%) patients, respectively.

Adjuvant oncology treatment of these patients was performed according to standard procedures 148 patients (74.7%) received radiotherapy. Adjuvant chemotherapy and hormone therapy as single modalities were used in 45 (22.7%) and 50 (25.2%) patients, respectively. Combined sequential chemotherapy plus hormone therapy was used in 78 (39.3%). Chemotherapy consisted of cyclophosphamide combined with other drugs, such as docetaxel and/or doxorubicin. Several antiestrogenic drugs were used as hormone therapy. Tamoxifen-based hormonotherapy was administered alone (83 patients, 43.7%) or in combination with other drugs, such as letrozole (12 patients, 6.3%), amongst others. Anastrozole alone was employed in 17 patients (8.9%). The rest of these patients did not receive adjuvant therapy on the basis of their age and/or health status.

Follow-up included a physical and clinical examination every 6 months, and breast imaging every 12 months. During follow-up, 37 (18.7%) patients died of the disease and 17 (8.6%) died of other causes. At present, 125 (63.1%) patients are alive and disease-free and 8 (4%) continue to have the illness. Follow-up data were not available for 11 patients (5.6%).

### Classification of tumors by immunohistochemistry (IHC)

3 μm sections were placed on slides and then deparaffinized, hydrated and treated to block endogenous peroxidase activity. After incubating with the appropriate primary antibodies (ER, PR, HER2, Ki-67) under the conditions summarized in Supplementary Table 4, the antibodies were developed using a Bond Polymer Refine Detection kit (Leica Biosystems, Newcastle, United Kingdom) and visualized with 3, 3′-diaminobenzidine (DAB). Slides were visualized and scored with the aid of the computer-assisted Ventana Image Analysis System (VIAS, Leica Biosystems). Tumors were classified with respect to the 2013 Saint Gallen consensus, for which a PR cut-point of ≥ 20% of cells was employed [3]. Subtypes identified were luminal A-like (32 cases, 16.4%), luminal B-like/HER2-negative (58 cases, 29.7%), luminal B-like/HER2-positive (47 cases, 24.1%), HER2-positive (19 cases, 9.7%), and triple-negative (39 cases, 20.0%) (Table 1). Additional markers (cytokeratin 5/6, cytokeratin 17, p63) were used to confirm the triple-negative subtype [47].

### DNA extraction from cell lines and tissue

As previously described, DNA was extracted from 0.5 × 10^6^ cells in the case of cell lines, while for tumors, it was obtained from a representative area with > 70% of tumoral cells fixed in 5-μm-thick paraffin sections selected by the pathologist [43] DNA concentration and quality were measured using a NanoDrop spectrophotometer ND-1000 (Thermo Scientific, Hanover Park, IL, USA). To assess DNA quality, the Δ-globin gene was amplified by PCR, as previously described [29].

### Methylation analysis of *RASSF1* and *RASSF2* genes

Methylation-specific PCR (MSP) of the *RASSF1* and *RASSF2* genes was performed in all cell lines and cases after sodium bisulfite modification of 1 μg of genomic DNA by Wizard DNA clean-up system kit (Promega Biotech Ibérica, Alcobendas, Spain) [15], using specific primers directed towards methylated and unmethylated sequences in a C1000 Thermal Cycler (Bio-Rad Laboratories, Hercules, CA, USA) [29]. DNA from lymphocytes treated *in vitro* with SssI methylase (New England Biolabs, Ipswich, MA, USA) and DNA from untreated lymphocytes were used as a positive and negative control, respectively. Bisulfite sequencing (BS) was performed in 10 tumors, 10 samples of normal breast tissue adjacent to tumors, and 4 samples of normal tissue from reduction mammoplasties to confirm *RASSF2* promoter methylation in 6 CpG positions of the gene, as previously described [43]. PCR products including this fragment were gel-purified and cloned into a pGEMT (Easy Vector System, Promega Biotech Ibérica, Madrid, Spain). DNA from 10 independent clones was randomly chosen, purified and sequenced in a 3100 Genetic Analyzer (Life Technologies Carlsbad, CA, USA). CpG methylation results were transformed into percentages.

### 5-azadC and trichostatin A treatment

To determine the effect of demethylation and acetylation on the expression of RASSF2 in cells, cell lines were treated with demethylating agent and acetylating agents after cultivation in triplicate in 6-well culture plates (Corning Life Sciences, Corning, NY, USA) and grown for 24 h. 5 mM of 5-azadC (Sigma-Aldrich) diluted in phosphate-buffered saline (PBS) (Life Technologies, Carlsbad, CA, USA) was then added to cultures for 72 h. Finally, 300 nM of trichostatin A (TSA) (Sigma-Aldrich) was added for an additional 24 h.

### qRT-PCR for RASSF2 expression in cell lines and cases

Real-time PCR experiments were performed in cell lines and cases to test for the presence of mRNA encoded by the *RASSF2* gene. RNA was isolated from control and treated cell lines with 5-azadC and TSA using the Ribopure kit including a DNAse I treatment (Ambion, Life Technologies, Carlsbad, CA, USA). Single-stranded cDNA obtained from 1 μg of RNA was synthesized using a Reverse Transcription kit (Life Technologies, Carlsbad, CA, USA). Real-time PCR reactions for analyzing the expression of the *RASSF2* gene were carried out using 100 ng of cDNA with TaqMan Universal PCR Master Mix, 1 × TaqMan gene expression assay (RASSF2: Hs00248129_m1) and GAPDH as the endogenous control (Life Technologies). In cases, five pairs of methylated luminal tumors and the corresponding normal tissue and five pairs of unmethylated triple-negative tumors and the corresponding normal cases were considered. RNA was extracted using the RecoverAll Nucleic Acid Isolation kit (Ambion, Life Technologies, Carlsbad, CA, USA). Single-stranded cDNA from 100 ng of RNA was synthesized using a High Capacity cDNA Reverse Transcription kit (Life Technologies, Carlsbad, CA, USA). Real-time PCR reactions from 40 ng of cDNA were performed as described above for cell lines.

All these experiments were performed in triplicate using an Applied Biosystems 7300 Sequence Detection System (Life Technologies), as described elsewhere [29]. The relative change in *RASSF2* expression in treatment compared with the control value and in luminal *versus* triple-negative cases was calculated by the delta-delta Ct method.

### Detection of RASSF2 expression in cell lines by immunofluorescence and in tumors by immunohistochemistry

To determine whether demethylating and acetylating treatments restore protein expression, immunofluorescence (IF) staining was performed in control and treated cell lines, and fluorescence intensity and protein location (nuclear and cytoplasmic) were analyzed. Cells were seeded on 6-well plates on top of a round glass slide. Combined treatment of 5-azadC and TSA was performed at the aforementioned concentrations. Cells were then fixed in fresh 4% paraformaldehyde (Merck, Darmstadt, Germany), permeabilized with 0.5% Triton 100-X for 30 min at room temperature, and blocked with 10% fetal bovine serum in PBS for 2 h. Subsequently, fixed cells were incubated with primary antibody diluted in PBS against RASSF2 (mouse, 1:150; Everest Biotech, Oxfordshire, OX, UK) at 4°C overnight. Slides were then incubated with Alexa Fluor 488-conjugated goat anti-rabbit IgG secondary antibody (Invitrogen, Life Technologies), diluted 1:200. Phalloidin (Alexa Fluor 594, 1:500; Invitrogen, Life Technologies) and DAPI Counterstain (Abbott Molecular, Des Plaines, IL, USA) were added for nucleus and cytoskeleton detection. Confocal microscopy was performed with a Leica TCS SP5 laser scanning microscope (with Acousto-Optical Beam Splitter) (Leica Biosystems) using in turn excitation wavelengths of 488 nm (for FITC) and 561 nm (for Texas Red). To measure fluorescence intensities of nuclear and cytoplasmic RASSF2, at least 100 cells of each condition group were captured using a 63 × HCX PL APO CS oil immersion objective 1.4 (NA). The average fluorescence intensities of the nucleus and cytoplasm, and the nucleo-cytoplasmic ratios were quantified with Definiens XD Software (Definiens, Munich, Germany) and pooled for each condition.

RASSF2 expression was evaluated by IHC in eight cases of each tumor subtype (four methylated and four unmethylated) under previously described conditions [43] The pattern of expression (nuclear, cytoplasmic) and the intensity of expression were evaluated by a pathologist who had no previous knowledge of the cases. The cases were scored as cytoplasmic or nuclear in the case if ≥ 50% positive cells were of cytoplasmic or nuclear pattern of expression, respectively. The intensity of expression was ascribed to one of three categories: 1, negative-weak (0–33% positive cells); 2, moderate (34–66% positive cells); 3, strong (67–100% positive cells), as previously described [47].

### Analysis of cell proliferation by Real-Time cell analysis (RTCA)

To study the effect of RASSF2 expression in breast cancer, luminal T-47D and triple-negative BT-549 cell lines were transduced using lentivectors, since these cells are the subtypes most differentiated with respect to their pathological and clinical characteristics. Expression plasmids based on pcDNA3.1/pDUAL encoding GFP (control) or RASSF2-FLAG were also constructed using previously established technology [38, 48] and purified by the Qiagen Plasmid Midi Kit. Lentivectors co-expressing the *RASSF2* and puromycin resistance genes were produced by the three-plasmid cotransfection method in 293T cells, and lentivector stocks were titrated by flow cytometry or quantitative PCR, as described elsewhere [20]. Cells were transduced with lentivector stocks at a transduction multiplicity of infection of 10. Transduced cells co-expressing RASSF2 and puromycin resistance were then selected with 2 μg puromycin/ml. RASSF2 expression and RASSF2 were detected by Western Blot using FLAG-specific antibodies

For the RTCA analysis 1.10^4^ control and transduced cells were seeded into 480 ul of media in E-plate L8 device (iCELLigence system, ACEA Biosciences, Inc., USA). Two replicates for each control cell line and four replicates for transduced cells were consider for each cell line (8 positions/cell line). The attachment, spreading and proliferation was monitored for 72 hours. The values obtained for cell index derives from cell sensor impedance, as previously described [49].

### Chemotherapy assays

Control and RASSF2-transduced cells were cultured before any other study in order to evaluate changes in growing behavior. Cell proliferation was assayed by counting proliferating cells with a hemocytometer. To compare the effect of commonly used chemotherapy drugs on cell survival in luminal and triple-negative cells related to RASSF2 expression, T-47D, BT-549, T-47D *RASSF2*, and BT-549 *RASSF2* cells were used. Following clinical criteria, luminal cells T-47D and T-47D RASSF2 were treated with docetaxel (10 nM and 50 nM), doxorubicin (250 nM and 800 nM), and a combination of the two (10 nM + 250 nM, 10 nM + 800 nM, 50 nM + 250 nM and 50 nM + 800 nM). Conversely, triple-negative cells (BT-549 and BT-549 RASSF2) were treated with paclitaxel (5 nM and 25 nM), cisplatin (10 μM and 20 μM) and a combination of both drugs (5 nM + 10 μM, 5 nM + 20 μM, 25 nM + 10 μM and 25 nM + 20 μM). 4 × 10^3^ of BT-549 cells and 15 × 10^3^ of T-47D cells/well were seeded in 96-well plates and grown overnight. Chemotherapy drugs were added for 72 h. Non-cell and PBS controls were included in all experiments. Four replicates per condition were established within the plate. The experiment was conducted independently three times. Cell survival was evaluated by an MTT assay following standard procedures [50].

### Statistical analysis

Demographic, clinical and pathological data were summarized as means (and standard deviations, SDs) or frequencies (and percentages), as appropriate. The presence of methylation of *RASSF2* in clones derived from each tumoral and normal sample was assessed graphically. Associations between percentage methylation and the patient age were measured by the Spearman coefficient. Associations between *RASSF1* and *RASSF2* hypermethylation, and pathological and clinical variables of this retrospective study were assessed with the X^2^ or Fisher's exact test. Differences in levels of RASSF2 expression detected by qRT-PCR between control and treated cell lines were evaluated by two-tailed unpaired Student's *t* tests. The 2^−ΔΔCt^ method was used to explore differences in the level of expression between control and treated cell lines and luminal and triple-negative tumors. To evaluate IF results, the mean values for each condition (control *vs.* treated, respectively) were compared using two-tailed unpaired *t*-tests, stratified by location (nucleus *vs.* cytoplasm) and considering the sum of the values for the two compartments. The same test was used for the analysis of the effects of RASSF2 re-expression analyzed by RTCA at different times (0, 24, 48, 72 hours).

Finally, the times between the dates of surgery and of recurrence or death were used to estimate, respectively, progression-free survival and overall survival. Kaplan–Meier plots and log-rank tests were used to examine the differences in survival time between patients with methylation-positive and methylation-negative tumors. Multivariate Cox proportional hazards regression models were fitted to test the independent contribution of each variable to the outcome after adjusting for other potential confounders. Hazard ratios (HRs) and 95% confidence intervals were used to estimate the effect of each variable on the outcome.

## SUPPLEMENTARY FIGURE AND TABLES


