# Supplementary material for: Differential involvement of RASSF2 hypermethylation in breast cancer subtypes and their prognosis
Source: Oncotarget. 2015 Jun 4;6(27):23944–58. doi: 10.18632/oncotarget.4062 (PMC4695163; doi:10.18632/oncotarget.4062)
Supplement: Supplementary file 1 [file oncotarget-06-23944-s001.pdf]

SUPPLEMENTARY FIGURE AND TABLES

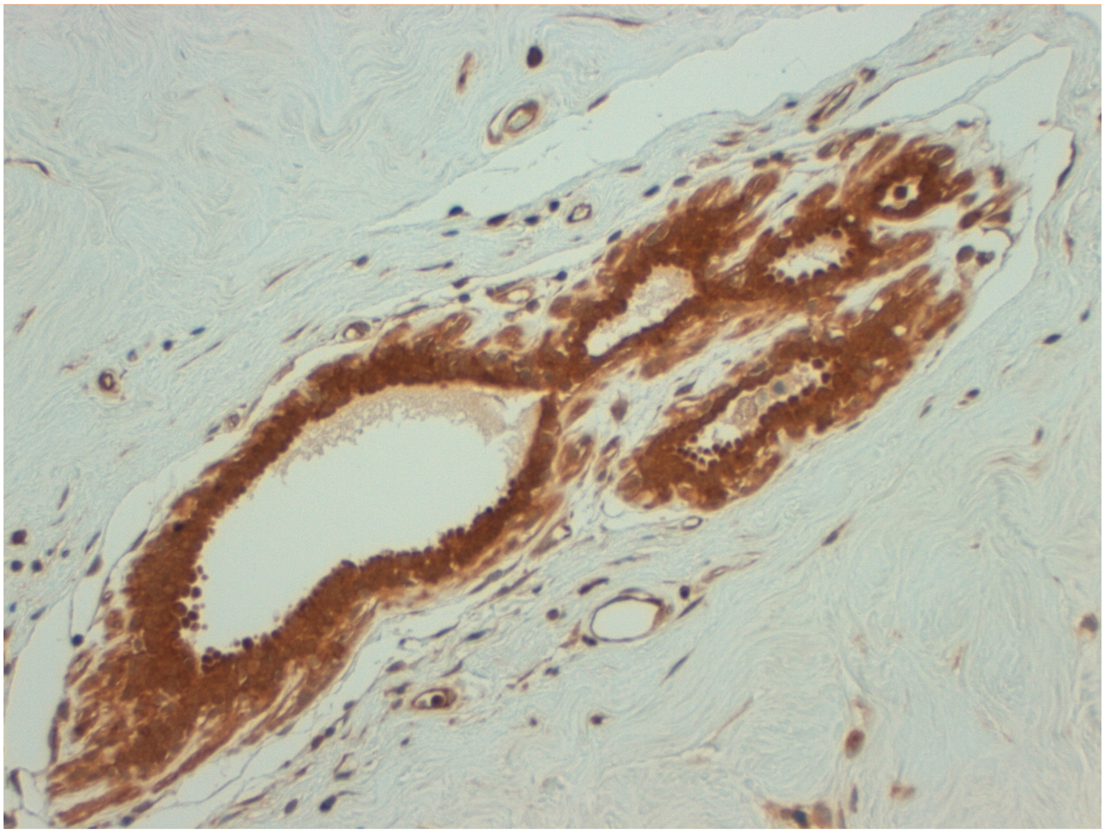

Supplementary Figure 1: IHC staining for RASSF2 expression, displaying featured intense expression in the cytoplasm of normal mammary ducts.

Supplementary Table 1: Average intensity values of RASSF2 expression obtained by immunofluorescence in treated compared with control cell lines

	Total		Cytoplasm		Nucleus	
**Cell line**	$\Delta_{tt\ vs.\ ctrl}$ **(95% CI)**	***p***	$\Delta_{tt\ vs.\ ctrl}$ **(95% CI)**	***p***	$\Delta_{tt\ vs.\ ctrl}$ **(95% CI)**	***p***
**BT-549**	4.77 (4.21, 5.32)	< 0.001	2.75 (2.19, 3.32)	< 0.001	6.78 (6.05, 7.52)	< 0.001
**SKBR3**	4.24 (3.81, 4,67)	< 0.001	3.36 (2.86, 3.86)	< 0.001	5.12 (4.47, 5.77)	< 0.001
**MCF 10A**	5.81 (4.75, 6.88)	< 0.001	5.47 (4.11, 6.84)	< 0.001	6.16 (4.61, 7.70)	< 0.001
**T-47D**	14.26 (13.20, 15.33)	< 0.001	8.71 (8.04, 9.38)	< 0.001	19.86 (18.52, 21.21)	< 0.001

Supplementary Table 2: Association of RASSF2 results obtained by Methylation-specific PCR and Immunohistochemistry (IHC)

	IHC scores*			***P***
	1	2	3	
**MSP results**				
**Methylated (*n* = 20)**	8 (40%)	6 (30%)	6 (30%)	0.061
**Not-methylated (*n* = 20)**	2 (10%)	6 (30%)	12 (60%)	

*Score: 1, negative-weak (0–33% positive cells); 2, moderate (34–66% positive cells); 3, strong (67–100% positive cells)

Supplementary Table 3: Effect of treatments on the survival of control and transduced cells

	Treatment							
	**Docetaxel**		**Doxorubicin**		**Combinations (Docetaxel + Doxorubicin)**			
	**10 nM**	**50 nM**	**250 nM**	**800 nM**	**10 nM + 250 nM**	**10 nM + 800 nM**	**50 nM + 250 nM**	**50 nM + 800 nM**
**T-47D**	48.3%	47.5%	60.7%	56.9%	61.5%	48.7%	64.2%	46.3%
**T-47D-RASSF2**	47.4%	47.7%	60.1%	49.4%	56.4%	41.9%	59.4%	45.3%

	Treatment							
	**Paclitaxel**		**Cisplatin**		**Combinations (Paclitaxel + Cisplatin)**			
	**5 nM**	**25 nM**	**10 µM**	**20 µM**	**5 nM + 10 µM**	**5 nM + 20 µM**	**25 nM + 10 µM**	**25 nM + 20 µM**
**BT-549**	52.3%	30.8%	62.7%	15.1%	57.1%	34.4%	41.8%	27.0%
**BT-549-RASSF2**	60.5%	34.3%	51.6%	6.8%	49.6%	31.6%	47.8%	23.3%

Supplementary Table 4: Panel of primary antibodies and conditions used in IHC

Marker	Clone	Dilution	*T*, incubation time	Commercial source
**ER***	SP1	pre-diluted	37°C, 32 min	Abcam, Cambridge, MA, USA
**PR***	1E2		37°C 28 min	Ventana Medical Systems, Roche, Mannheim, Germany
**HER2***	4B5		42°C, 32 min	Roche, Mannheim, Germany
**Ki-67***	30-9		37°C, 20 min	Roche, Mannheim, Germany
**Cytokeratin 5/6****	D5/16B4		42°C, 32 min	Roche, Mannheim, Germany
**Cytokeratin 17****	E3		R.T., 28 min	Roche, Mannheim, Germany
**p63****	BC4A4		R.T., 35 min	Menarini, Barcelona, Spain
**P53**	Bp53-11		37°C, 36 min	Roche, Mannheim, Germany
**RASSF2**	EPR6621	1:100	R.T., 60 min	Epitomics, San Diego, CA, USA

min: minutes; R.T.: room temperature.
*Antibody used for classifying tumors into breast cancer subtypes (luminal A, luminal B, luminal-HER2, HER2, basal)
**Antibody used for basal subtype
